# Papillary tumor of the pineal region accompanied by Parinaud’s syndrome: magnetic resonance imaging findings

**DOI:** 10.1590/0100-3984.2016.0229

**Published:** 2018

**Authors:** Bruno Niemeyer de Freitas Ribeiro, Bernardo Carvalho Muniz, Nina Ventura, Emerson Leandro Gasparetto, Edson Marchiori

**Affiliations:** 1 Instituto Estadual do Cérebro Paulo Niemeyer and Universidade Federal do Rio de Janeiro (UFRJ), Rio de Janeiro, RJ, Brazil.; 2 Instituto Estadual do Cérebro Paulo Niemeyer, Rio de Janeiro, RJ, Brazil.; 3 Universidade Federal do Rio de Janeiro (UFRJ), Rio de Janeiro, RJ, Brazil.

*Dear Editor,*

A 22-year-old male patient presented with a nonpulsatile, diffuse headache of moderate-intensity, with no aura or other associated symptoms. In the neurological exam, he presented paralysis of the vertical conjugate gaze with fixed downward glance, bilateral eyelid retraction, insufficiency of ocular convergence, pupils nonreactive to light, and preserved pupillary reaction to accommodation, characterizing Parinaud’s syndrome. Magnetic resonance imaging (MRI) showed an expansive lesion in the pineal region, with a discrete hyperintense signal in T1-weighted sequences and isointense in T2-weighted sequences, with cystic areas of diffusion, a discrete hyperintense signal in the diffusion, and marked gadolinium enhancement (Figure 1). The lesion caused compression of the cerebral aqueduct and dorsal midbrain, as well as causing hydrocephalus. Histopathological analysis demonstrated a papillary neoplasm composed of cuboidal cells, with an epithelial appearance, arranged on fibroconnective stromata, with evident vascularization, and mitotic activity (4 mitotic figures per 10 high-power fields). Immunohistochemical analysis showed marked positivity for cytokeratins and for S-100 protein, together with negativity for neurofilament proteins. These findings are consistent with a papillary tumor of the pineal region (PTPR).

**Figure 1:**
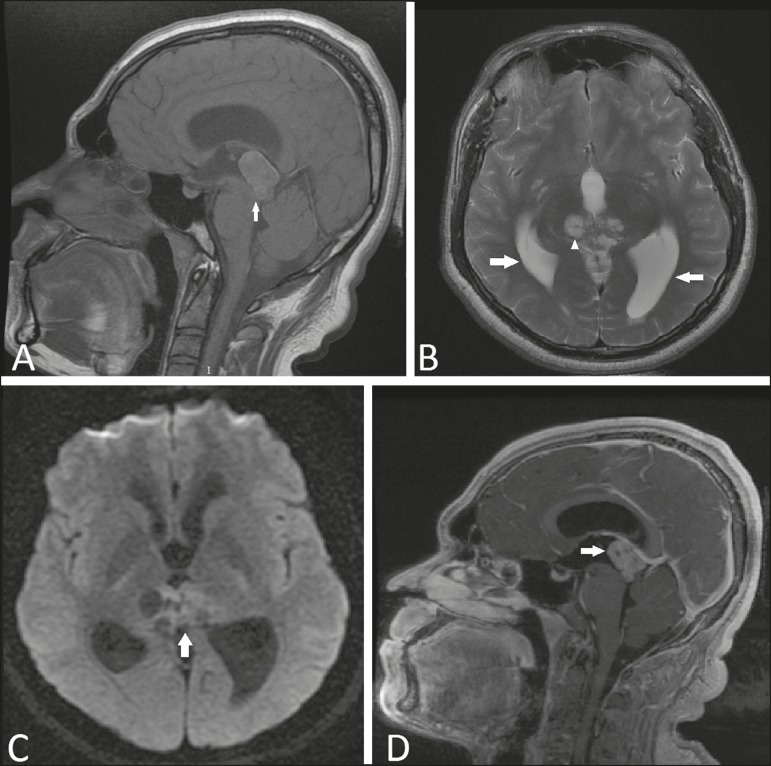
Magnetic resonance imaging. **A:** Non-contrast-enhanced sagittal T1-weighted sequence showing an expansile lesion in the pineal region compressing the dorsal midbrain and presenting a predominance of high signal intensity (arrow). **B:** Axial T2- weighted sequence showing cystic images within the lesion (arrowhead). Note also the increase in the dimensions of the supratentorial ventricular system (arrows). **C:** Functional diffusion- weighted sequence, axial section, showing discrete hyperintensity. **D:** Gadolinium contrast-enhanced sagittal T1-weighted sequence showing heterogeneous enhancement.

The role of MRI in the diagnosis of brain tumors has been expanding^(^^1^^-^^3^^)^. The World Health Organization classifies PTPR as a grade II or III tumor. It is rare, fewer than 200 cases having been reported. The mean age at onset is 35 years, and PTPR has no predilection for either gender^(^^4^^)^. Its origin is uncertain, the most widely accepted hypothesis is that it originates from ependymal cells of the subcommissural organ^(^^4^^,^^5^^)^. Histologically, PTPR is characterized by the presence of epithelial and papillary aspect structures with high cellularity and moderate-to-high mitotic activity^(^^4^^-^^6^^)^. It can cause headache, due to obstructive hydrocephalus, and Parinaud’s syndrome^(^^7^^)^, due to compression of the dorsal midbrain, specifically the periaqueductal region^(^^8^^)^.

On MRI, PTPR typically presents as a heterogeneous, well-circumscribed mass in the pineal region, containing cystic areas, without calcifications or hemorrhages. Classically, it is described as showing a hyperintense signal in T1-weighted sequences, as observed in our case, the high signal intensity potentially being related to the high protein content of the lesion ^(^^9^^,^^10^^)^. After intravenous administration of gadolinium, moderate heterogeneous enhancement is observed. Dissemination into the cerebrospinal fluid, although rare, occurs in up to 7% of cases^(^^4^^,^^7^^)^. Advanced MRI sequences can reveal signs of hypoperfusion, whereas proton spectroscopy can show increases in the peaks of choline, lactate, and myo-inositol, as well as a decrease in the N-acetyl-aspartate peak^(^^11^^)^.

The differential diagnosis of lesions in the pineal region is broad, including germinoma, ependymoma, meningioma, pineocytoma, pineoblastoma, and glioma, although those tumors rarely present a hyperintense signal in T1-weighted sequences^(^^10^^,^^11^^)^.

The treatment of choice is surgical resection, there being no proven benefits of the use of radiotherapy or chemotherapy^(^^11^^)^. Partial resection and tumors with higher mitotic and proliferative activity (high Ki-67 expression) tend to be related to a poor prognosis and to recurrence, which is reported in up to 72% of cases^(^^4^^,^^11^^,^^12^^)^.

In conclusion, Parinaud’s syndrome is a warning sign of the possibility of expansile processes in the pineal region. Albeit rare, the diagnosis of PTPR should be remembered among the hypotheses, especially when there is a hyperintense signal in a T1-weighted sequence.

## References

[1] Queiroz RM, Abud LG, Abud TG (2017). Burkitt-like lymphoma of the brain mimicking an intraventricular
colloid cyst. Radiol Bras.

[2] Liaffa B, Noro F, Bahia PRV (2017). Dural fistula with bilateral arterial supply, mimicking a
brainstem tumor. Radiol Bras.

[3] Sharma R, Gupta P, Mahajan M (2016). Giant nontraumatic intradiploic arachnoid cyst in a young
male. Radiol Bras.

[4] Louis DN, Ohgaki H, Wiestler OD (2016). WHO classification of tumours of the central nervous system.

[5] Kamamoto D, Sasaki H, Ohara K (2016). A case of papillary tumor of the pineal region with a long
clinical history: molecular characterization and therapeutic consideration
with review of the literature. Brain Tumor Pathol.

[6] Jiménez-Heffernan JA, Bárcena C, Gordillo C (2016). Cytologic features of papillary tumor of the pineal region: a
case report showing tigroid background. Diagn Cytopathol.

[7] Fauchon F, Hasselblatt M, Jouvet A (2013). Role of surgery, radiotherapy and chemotherapy in papillary
tumors of the pineal region: a multicenter study. J Neurooncol.

[8] Shields M, Sinkar S, Chan W (2017). Parinaud syndrome: a 25-year (1991-2016) review of 40 consecutive
adult cases. Acta Ophthalmol.

[9] Cerase A, Vallone IM, Di Pietro G (2009). Neuroradiological follow-up of the growth of papillary tumor of
the pineal region: a case report. J Neurooncol.

[10] Chang AH, Fuller GN, Debnam JM (2008). MR imaging of papillary tumor of the pineal
region. AJNR Am J Neuroradiol.

[11] Vaghela V, Radhakrishnan N, Radhakrishnan VV (2010). Advanced magnetic resonance imaging with histopathological
correlation in papillary tumor of pineal region: report of a case and review
of literature. Neurol India.

[12] Fèvre-Montange M, Hasselblatt M, Figarella-Branger D (2006). Prognosis and histopathologic features in papillary tumors of the
pineal region: a retrospective multicenter study of 31 cases. J Neuropathol Exp Neurol.

